# Building a One Health mobile laboratory workforce: strengthening public health preparedness across Europe and Africa

**DOI:** 10.1186/s12960-026-01097-5

**Published:** 2026-09-03

**Authors:** Aryse Martins Melo, Beate Becker-Ziaja, Markus Keller, Duncan Sangale, Dewi Ismajani Puradiredja, Theresa Habermann, Juliane Bönecke, Alexandru Tomazatos, Johannes R. Peham, Birgit Michalek, Andry Aasamäe, Georg G. Duscher, Lisa Winkelmayer, Denise Framke, Monique-Theres Schranz, Daniela Gleißner, Martin Groschup, Fereshteh Haghighi, Patrick Slowikowski, Katrin Schwabe, Lida Politi, Kassiani Mellou, Anastasia Flountzi, Dafni Dimitriadou, Ioanna Spiliopoulou, Grigoris Spanakos, Andriani Varela, Ramadhani Libenaga, Mariagoreth Beda, Edna Mgimba, Andrew Ugolole, Jackson Rashidi, Anselmi Munga, Proscovia Kagaruki, Shabani Motto, Ambele Mwafulango, Nyambura Moremi, Peter Mkama, Jürgen May, Muna Affara, Florian Gehre

**Affiliations:** 1 Infectious Diseases Epidemiology Department, Bernhard Nocht Institute for Tropical Medicine (BNITM), Hamburg, Germany; 2 Service Unit Mobile Laboratory, Bernhard Nocht Institute for Tropical Medicine (BNITM), Hamburg, Germany; 3 Institute of Novel and Emerging Infectious Diseases, Friedrich-Loeffler-Institut (FLI), Federal Research Institute for Animal Health, Greifswald-Insel Riems, Germany; 4 Interdisciplinary Academy of Competence and Education for Global Health, Centre for Education and Training, Bernhard-Nocht-Institute for Tropical Medicine (BNITM), Hamburg, Germany; 5 Austrian Institute of Technology (AIT), Center for Health and Bioresources, Competence Unit Molecular Diagnostics, Vienna, Austria; 6 MDSC Systems OÜ, Tallinn, Estonia; 7 Austrian Agency for Health and Food Safety (AGES), Moedling, Austria; 8 Ethnikos Organismos Dimosias Ygeias (EODY)/National Public Health Organization (NPHO), Athens, Greece; 9 National Public Health Laboratory (NPHL), Dar Es Salaam, Tanzania; 10 National Environment Management Council (NEMC), Dar es Salaam, Tanzania; 11 Tanzania Agricultural Research Institute (TARI), Dar es Salaam, Tanzania; 12 Tanzania Wildlife Research Institute (TAWIRI), Arusha, Tanzania; 13 Tanzania Veterinary Laboratory Agency (TVLA), Dar es Salaam, Tanzania; 14 German Center for Infection Research (DZIF), Partner Site Hamburg-Lübeck-Borstel-Riems, Hamburg, Germany; 15 Tropical Medicine II, University Medical Center Hamburg-Eppendorf (UKE), Hamburg, Germany

**Keywords:** Training-of-trainers, Health care professionals, Capacity building, Preparedness, Emergency response, One Health

## Abstract

The emergence and re-emergence of infectious diseases remain a major public health threat in Europe. The Horizon Europe-funded MOBILISE project addresses this challenge by establishing a One Health Mobile Laboratory capable of handling risk group 3 and 4 pathogens, while strengthening outbreak preparedness through targeted capacity-building activities and the development of a rapid-response expert network. A key component of the project is a training-of-trainers (ToT) model, which enables participants to perform critical tasks in the field and transfer expertise within their home countries. This approach expands the pool of skilled professionals and strengthens collective preparedness. A descriptive mixed-methods programme evaluation, including pre- and post-test assessments, was conducted to assess the ToT model's implementation. In the first phase, two laboratory experts from each partner country were selected. They completed online courses, followed by a two-week in-person training at the Bernhard Nocht Institute for Tropical Medicine in Hamburg, Germany. Training covered biosafety, molecular diagnostics, sequencing, and mobile laboratory operation. The programme concluded with four field missions, allowing participants to apply their skills and train new cohorts. The evaluation also highlighted challenges related to retaining trained experts within the expert pool over time. This structured approach enhances outbreak preparedness, fosters cross-border collaboration, and strengthens the resilience of the health system.

## Introduction

Climate change is contributing to the emergence and re-emergence of infectious diseases [1]. According to the *European State of the Climate*, 2024 was the warmest year on record for both Europe and the globe [2]. Rising temperatures and increased international travel have contributes to a higher risk of importing and locally transmitting infectious diseases such as malaria, viral haemorrhagic fevers (Ebola and Marburg virus disease), chikungunya, dengue, West Nile, and Crimean-Congo haemorrhagic fever [3]. In this context, the One Health approach is essential to improve preparedness and response to emerging zoonotic threats [4].

Facing this public health threat requires two essential components: (i) robust laboratory infrastructure and high-containment biosafety facilities capable of handling risk group 3 and 4 pathogens for diagnosing and managing (re)-emerging infectious diseases; and (ii) a skilled workforce to operate such facilities and safely handle and diagnose highly infectious pathogens. Although the number of stationary BSL-3 and BSL-4 laboratories is increasing worldwide, many countries still do not have access to such infrastructure, lack adequate biosafety protocols [5] or do not have the required laboratory operators [6, 7]. In the context of infectious disease outbreaks, the effective and safe operation of high-containment laboratories critically depends on the presence of highly qualified personnel. Strengthening the health workforce is increasingly recognised as a cornerstone of global health security, as preparedness and response capacities rely not only on infrastructure and technologies but also on trained personnel capable of safely performing diagnostics, surveillance, and outbreak response activities [8]. Personnel competence is defined by an integrated combination of formal qualifications, specialised training, practical skills, and relevant professional experience [9]. Recent international guidance for rapid response mobile laboratories (RRML) further emphasises that workforce preparedness should rely on continuous competency development, regular assessment, and the maintenance of trained personnel rosters to ensure rapid and interoperable deployment during health emergencies [10, 11].

In RRMLs, where operational constraints and field conditions add layers of complexity, targeted training programmes and structured mentorship are essential to ensure proficiency in assigned responsibilities. This approach guarantees the reliable execution of diagnostic and research activities, as well as strict adherence to biosafety and biosecurity standards under dynamic and challenging conditions in the field [11].

The MOBILISE project addresses both shortcomings: it developed a novel One Health mobile laboratory concept capable of handling risk group 3 and 4 pathogens even in remote areas, and also established a European and African pool of laboratory experts capable of diagnosing epidemic-prone pathogens and operating the mobile laboratory.

Within this context, the research question addressed in this study is: Can a structured One Health training-of-trainers (ToT) programme effectively prepare a transnational workforce for mobile laboratory deployment and outbreak response? To answer the question, this study describes the design and implementation of the ToT programme and evaluates, through four field simulation and outbreak response missions conducted in Europe and Africa, its contribution to building a sustainable and interoperable network of trained laboratory experts.

## Methods

### Study design

This study employed a mixed-methods, descriptive programme evaluation design to assess the development and implementation of the MOBILISE One Health training-of-trainers (ToT) programme. Knowledge acquisition during the online training module and in-person training were assessed using a single-group pretest—posttest design, without a control group. The subsequent field validation phase was evaluated descriptively, drawing on structured observation and participant performance during the field missions, prioritising the competency-based assessment principles [12].

### Training-of-trainers programme

The training-of-trainers (ToT) methodology was adopted for this project, as it is grounded on the premise that the effective dissemination of knowledge and the sustainable strengthening of technical capacities are achievable when well-prepared specialists assume the role of mentors within their own institutions [13, 14]. According to this principle, upon completion of the initial training programme, trainees transition into trainers themselves, enabling them to build local capacities by training colleagues under the guidance and supervision of experienced mentors.

### Expert selection criteria

The selection of participants for the ToT programme was guided by specific criteria which ensured both technical competence and the potential for knowledge dissemination within their respective institutions. Candidates were required to have a background in molecular laboratory operations, such as nucleic acid extraction, RT-qPCR, serology and (ideally) next generation sequencing. Each partner institution nominated two individuals who demonstrated leadership potential to become trainers and a strong commitment to robust laboratory principles.

For this training initiative, experts were selected from the following institutions: Austrian Agency for Health and Food Safety (AGES, Austria), the Friedrich-Loeffler-Institute (FLI, Germany), the National Public Health Organization (EODY, Greece), and the National Public Health Laboratory (NPHL, Tanzania).

### Training structure

To structure the programme efficiently and maximize learning outcomes, a phased approach was adopted. To design the training curriculum, an assessment of end-user training needs was systematically conducted in collaboration with partner institutions, ensuring that the content was directly aligned with the practical requirements and operational priorities of the MOBILISE mobile laboratory. The curriculum was composed of three key stages: an initial online training module, an intensive in-person training experience, and comprehensive field validation exercises designed to facilitate and strengthen real-world operations. This multi-tiered methodology was intended to build robust theoretical understanding and to foster hands-on proficiency and adaptability in diverse operational environments.

### Online training

The online training was hosted on the German Online Platform for Global Health Protection (GO4GHP; https://go4ghp.de). This training laid the foundational knowledge required for high-containment laboratory work during health emergency response, covering essential topics in emergency response during infectious diseases outbreaks, one health approach, biosafety, biosecurity, the principles of pathogen detection and containment as well as next-generation sequencing. This remote phase enabled participants to engage with instructional materials, case studies, and interactive assessments at their own pace, ensuring a uniform baseline of understanding before progressing to more advanced modules.

### In-person training programme

The subsequent two-week, in-person training programme took place in February 2025 at BNITM. The programme provided an immersive One Health-oriented environment, where participants engaged directly with state-of-the-art laboratory equipment and received hands-on training in all workflows and protocols relevant to field operations. Under the guidance of experienced mentors, trainees practiced critical procedures and developed the practical competencies essential for the safe and effective operation of mobile laboratories handling high-risk pathogens. This included commissioning and decommissioning the MOBILISE truck laboratory, as well as setting up the lab for field deployment.

### Field validation and knowledge transfer

Finally, for the field validation, participants deployed the MOBILISE mobile laboratory in simulated outbreak environments, allowing them to apply their skills in real-world settings and adapt to the logistical and technical challenges unique to fieldwork. The field validation phase lasted three weeks in each region and was implemented in the participants’ respective countries: Austria, Germany, Greece, and Tanzania. The first week focused on a refresher training for the trainers and the initiation of knowledge transfer to local colleagues, followed by two weeks of intensive field simulations where participants practiced the complete workflows under realistic operational conditions.

### Assessment of competency and operational preparedness

Knowledge acquisition was evaluated using pre- and post-tests administered immediately before and after the online and in-person training components.

Competency during the field validation phase was assessed through continuous, mentorship-based observation during field simulations, without the use of a structured checklist. Experienced mentors who delivered the in-person training accompanied participants throughout the field missions and evaluated technical performance, adherence to Standard Operating Procedures (SOPs), teamwork, problem-solving, and operational adaptability, providing real-time feedback and troubleshooting support.

Operational preparedness was assessed through performance in practical exercises and field missions, adherence to SOPs, compliance with biosafety and biosecurity requirements, and diagnostic turnaround time (TAT), defined as the time from sample receipt to reporting of molecular diagnostic results. TAT was used as a key operational indicator of field laboratory performance and deployment readiness within the RRML context, with an intended target of achieving results within a maximum timeframe of 8 h [13].

## Results

### Identification of end user needs

The needs assessment conducted with partner institutions highlighted several critical areas for capacity building to ensure the effective deployment of the MOBILISE mobile laboratory. A key priority identified was training in the safe handling and inactivation of risk group 4 pathogens within a glovebox. Although expertise in sample collection and subsampling was already available, partners required additional skills tailored to the specific operational context of the mobile laboratory. Further gaps were noted in sample handling and molecular techniques: while veterinary sample processing and arboviral PCR diagnostics were partly established, training was considered essential to cover procedures not routinely performed in partner laboratories, particularly those aligned with the SOPs of the mobile laboratory. The assessment also underscored the importance of expanding capacities in specialised arboviral PCR diagnostics and simplified pipelines for whole genome sequencing. Operational aspects including laboratory setup and decommissioning, emergency response, waste management, infrastructure maintenance, and biosafety measures were also identified as essential training domains. Finally, the assessment pointed to the necessity of equipping staff with competencies in the use of a customised Laboratory Information Management System (LIMS) to enable efficient sample management and documentation. A summary of the training needs assessment can be found in Table 1.

**Table 1:** Summary of training needs within the MOBILISE consortium partners

MOBILISE lab procedure	Training requirement
Sample reception Risk Group 4 pathogens	YES (All partners)
Inactivation of Risk Group 4 pathogens	YES (All partners)
Veterinary sample reception and subsampling	YES (Some partners)
Triple packaging of infectious substances	No
RNA/DNA extraction	No
Arboviral PCR diagnostics	YES (Some partners)
One Health sequencing	YES (Partners have sequencing capacity, but training required in specific workflow)
One Health genomic analysis	YES (Partners have sequencing capacity, but training required in specific pipelines)
LIMS	YES (All partners)
Mobile lab operations (set up and decommissioning, waste management, equipment maintenance, PPE, etc.)	YES (Some partners)

### Online training

The online training curriculum consisted of five modules: health emergency response, One Health approach, diagnostics—including virus biology and molecular diagnostic methods, introduction to next-generation sequencing (NGS), and biosafety and biosecurity. A total of eight experts, comprising two representatives from each partner country, were enrolled in the online training. The assessment results showed that participants already possessed a solid baseline of knowledge prior to the course, with further improvements observed after training. Pre-training scores ranged from 4 to 8, with a mean of 6.625, whereas post-training scores ranged from 7 to 10, with a mean of 8.857 (maximum score: 10).

(Fig. 1).

**Fig. 1:**
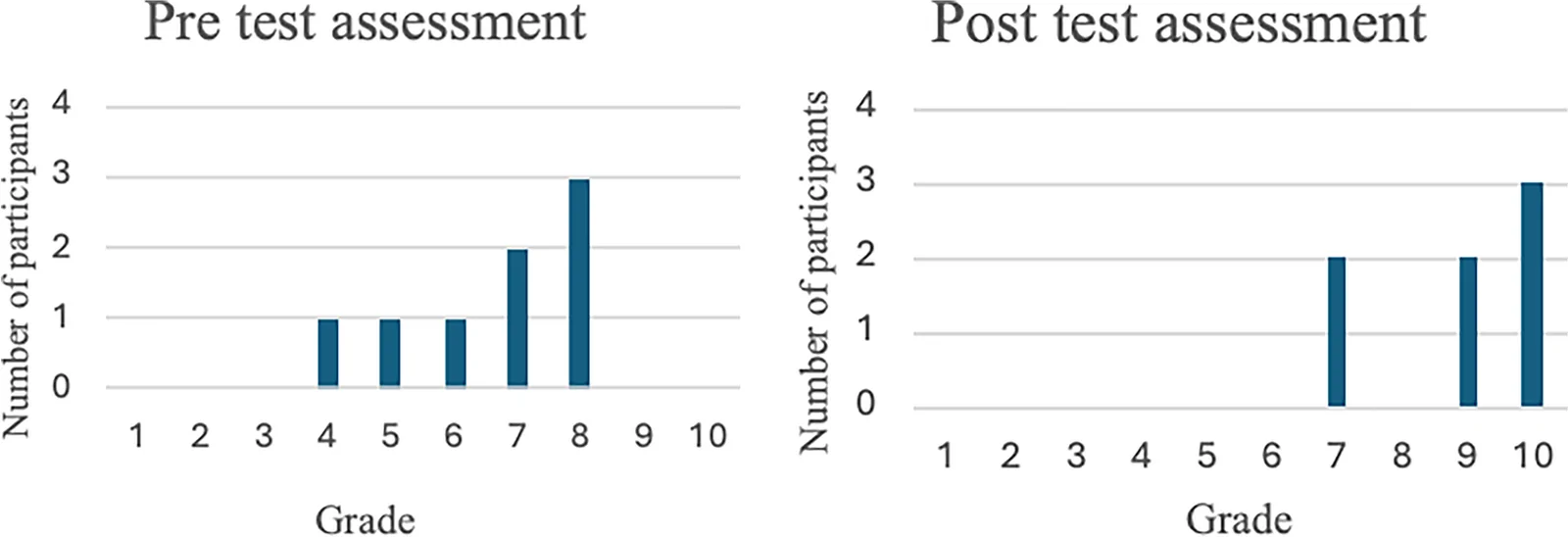
Pre- and post-training test scores of participants. Participants showed a mean score of 6.625 (*n*=8 participants) before training (“pre-test”) and a mean score of 8.857 (*n*=7 participants) following the training (“post-test”). During the second test one of the trainees did not participate

### In-person training programme

The in-person training provided participants with integrated, hands-on experience in all operational aspects of the MOBILISE mobile laboratory. Trainees gained competencies in laboratory setup and decommissioning, biosafety procedures, and the safe handling of high-risk pathogens within a glovebox. The programme also covered One Health sample collection and processing, nucleic acid extraction, arboviral RT-qPCR diagnostics, and the use of automated microbiology platforms. Additional modules included Next-Generation Sequencing workflows, hazardous waste management, and the use of a customised Laboratory Information Management System (LIMS) to support data integrity and workflow efficiency. A total of eight experts, comprising two representatives from each partner country, were enrolled in the in-person training. By the end of the programme, participants demonstrated the ability to safely and effectively operate the mobile laboratory, from initial setup through advanced diagnostics to final decontamination and shutdown. Pre-training scores ranged from 9 to 13, with a mean of 10.85, whereas post-training scores ranged from 10 to 13 with a mean of 11.28 (maximum score: 13).

### Field validation and knowledge transfer

**Fig. 2:**
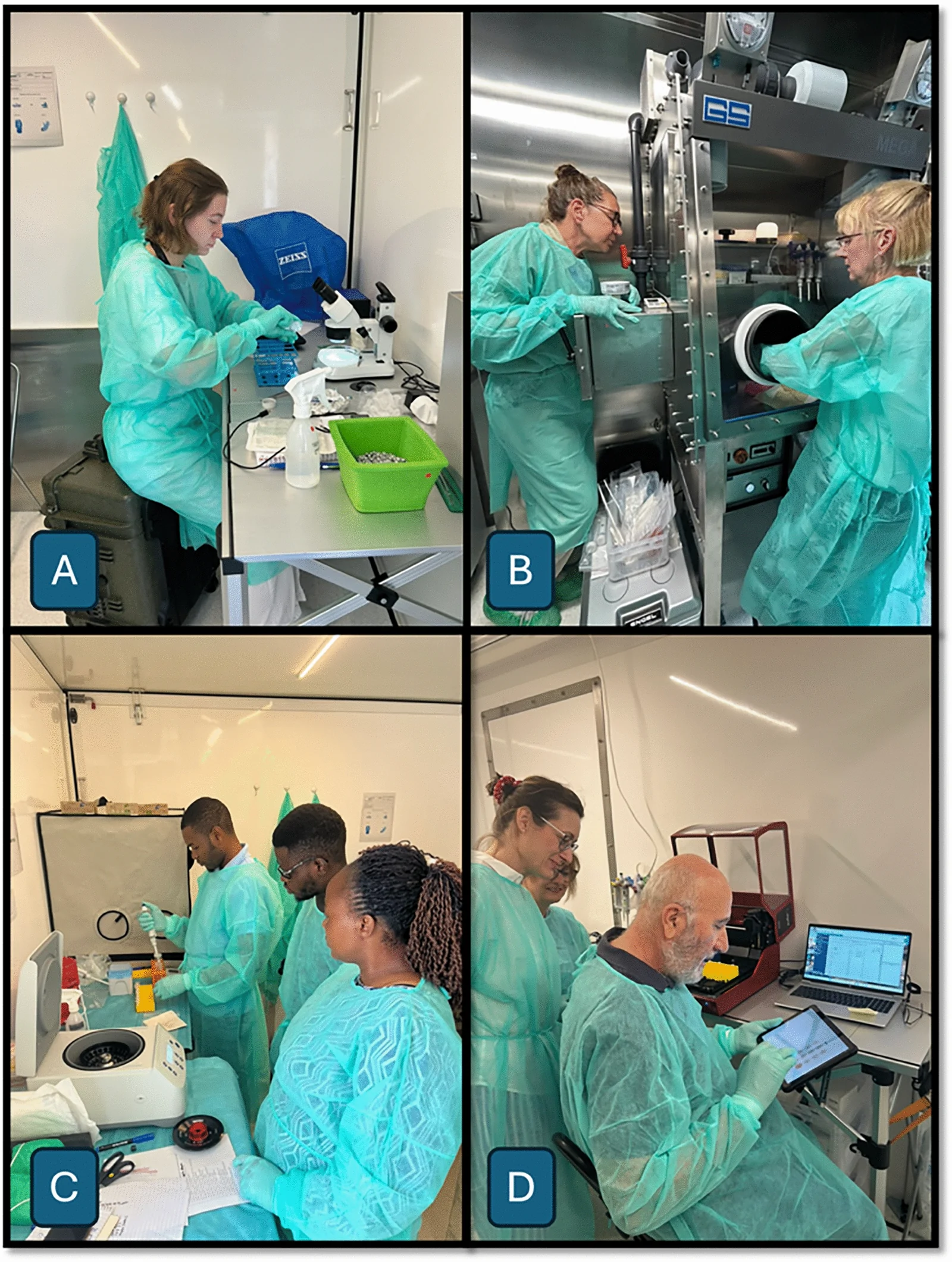
MOBILISE pool of trained operators performing standardised procedures according to established SOPs across different field missions. **A** Tick identification during the Austrian field mission; **B** Sample inactivation within the glovebox during the German field mission; **C** Nucleic acid extraction during the Tanzanian field mission; **D** PCR setup and LIMS operation during the Greek field mission

The final step of the comprehensive MOBILISE training program consisted of field validation of both the mobile laboratory and ToTs, consolidating and transferring the acquired knowledge. During the field missions, one additional expert was trained in Austria, three in Greece, one in Germany, and eight in Tanzania. By the end of the four field missions, a total of 21 experts from Europe and Africa had been trained for the full operation of the MOBILISE mobile laboratory. The trained experts were able to apply the knowledge gained throughout the programme to various operational tasks within the MOBILISE framework, consistently following the established SOPs. The systematic application of SOPs ensures that all activities conducted in the MOBILISE mobile laboratory adhere to the same standards, regardless of the country in which the operations take place (Fig. 2).

#### Field mission 1: Crimean-Congo haemorrhagic fever virus simulation in Moedling, Austria, April/May 2025

The first field deployment of the MOBILISE mobile laboratory was conducted from April 21 to May 16, 2025, in Moedling, Austria, centring on a simulated outbreak scenario involving the Crimean-Congo haemorrhagic fever virus (CCHFV). The operational deployment of the mobile laboratory enabled the trained personnel to execute the complete diagnostic workflow. On simulation day, the laboratory team received 6× cattle blood samples, *Ixodes ricinus* and *Hyalomma marginatum* ticks, for species identification and pooling, and 4 human samples. These human, animal, and vector samples (12 in total) were inactivated within a glovebox, nucleic acids were extracted, RT-qPCRs performed, and results reported to AGES with the help of a customized LIMS system. The team successfully completed all steps with a diagnostic TAT of six hours, demonstrating training effectiveness and the preparedness of the mobile laboratory to respond efficiently to outbreak scenarios, as detailed in Table 2.

**Table 2:** Diagnostic workflow and timeline of the MOBILISE field mission in Austria, May 2025

Time	Activity
07:04	Sample arrived at the sample reception area
07:25	Sample reception completed
07:26	Samples brought into the BSL-3 anteroom and imported into the BSL-3 lab
07:26	Operator 1 began preparations for tick identification
07:27	Operator 2 entered BSL-3 room
07:31	Operator 2 imported blood samples into the glovebox
07:31	Operator 1 began tick identification
07:47	Blood samples incubated at room temperature to allow blood plasma separation. Operator 3 started entering data into the LIMS
07:55	Operator 1 began tick homogenization
08:14	Tick homogenization completed
08:25	Operator 2 began work in the glovebox
08:46	Operator 3 completed LIMS data entry
09:20	Sample inactivation complete, inactivated samples exported to RNA extraction area
09:25	Operator 2 began RNA extraction
10:15	RNA extraction completed
10:40	Operator 1 began PCR setup
12:20	PCR analysis started
12:44	PCR analysis completed
12:45	Supervisor validation started
13:29	Results sent to DSS

#### Field mission 2: Avian influenza virus in Isle of Riems, Germany, June 2025

The German field mission of the MOBILISE training programme took place from May 22 to June 20, 2025, at the Isle of Riems to assess the mobile laboratory’s performance in a simulated outbreak of avian influenza virus (AIV). At the time of the mission, one of the ToTs was no longer part of the team. Previously trained lab experts consolidated their skills, whilst one new expert received targeted training during the field mission.

The field mission served to validate laboratory operations, including sample processing, RT-qPCR testing, and the use of a dedicated Laboratory Information Management System (LIMS). 45 environmental samples of bird faeces were collected and processed under appropriate biosafety conditions. The team successfully completed all diagnostic steps, and all the results were further confirmed under quality management conditions using standard diagnostic instruments at FLI, demonstrating both the effectiveness of the training program and the operational readiness of the mobile laboratory.

#### Field mission 3: West Nile virus in Nea Madytos, Greece, July 2025

The Greek field mission of the MOBILISE training programme took place from June 30 to July 18, 2025. At the time of the mission, ToTs previously trained in Hamburg were no longer part of the team. Consequently, an entirely new team of lab experts had to be trained from scratch to carry out the field deployment. The first week was dedicated to intensive training in Athens, covering all essential laboratory procedures, high-containment handling, and operational workflows.

Following the training phase, the mobile laboratory was deployed to Nea Madytos in northern Greece, a region with a documented history of West Nile virus (WNV) outbreaks. During the mission, the lab experts successfully executed the full diagnostic workflow, achieving a TAT from sample reception to results reporting of approximately 4.5 h (Table 3). The deployment demonstrated both the adaptability of the MOBILISE training system to rebuild a fully functional team and the operational readiness of the mobile laboratory to respond efficiently to high-risk vector-borne disease scenarios.

**Table 3:** Diagnostic workflow and timeline of the MOBILISE field mission in Greece, July 2025

Time	Activity
13:00	Samples arrived at MOBILISE
13:30	Last sample arrived at MOBILISE
13:15	Start sample inactivation in the class II biosafety cabinet (BSCII)
14:00	Sample inactivation complete, inactivated samples exported to RNA extraction area
15:00	RNA extraction completed
15:00	PCR setup
16:35	PCR analysis started
17:00	PCR analysis completed
17:00	Supervisor validation started
17:30	Reports sent to central database at EODY

#### Field mission 4: Rift Valley fever virus in Kigoma region, Tanzania, October 2025

The last field mission of the MOBILISE training programme took place from October 20 to November 7, 2025, in the Kasulu district of the Kigoma region. The mission aimed to support Rift Valley fever virus (RVFV) surveillance through rapid on-site diagnosis in both humans and animals. The mobile laboratory was stationed next to the Kasulu District Hospital, serving as the operational base for integrated One Health activities.

The field team comprised two experts from BNITM, two trained trainers (ToTs) from the Tanzanian National Public Health Laboratory (NPHL), and eight multidisciplinary trainees representing the human, animal, and environmental health sectors. Training and diagnostic activities were conducted simultaneously, reproducing real outbreak-response conditions and ensuring hands-on experience across all laboratory workstations, including sample inactivation, serology, nucleic acid extraction, PCR analysis, as well as using the LIMS to manage and document all lab work. Samples were collected from hospitals and farms across the Kigoma region. A total of 136 samples were analysed, including 74 human and 62 animal samples. The TAT to process 20 samples was 5.5 h on average for RT-qPCR and 27 h for ELISA, including a 24-h incubation period.

## Discussion

The establishment of a One Health expert pool through the MOBILISE training programme significantly strengthens rapid outbreak response capacities. The results from the field missions in Austria, Germany, Greece and Tanzania, involving different teams and operational contexts, provide a repeated real-world demonstration of the programme’s effectiveness in preparing laboratory experts to safely and efficiently operate high-containment mobile laboratories. This multi-site, multi-team evaluation effectively represents four independent validations of training outcomes across two continents.

Turnaround times for molecular diagnostics ranged from approximately 4.5 to 6 h, indicating stable operational performance across field settings and consistency with established RRML deployments. An operational target of ≤8 h was defined based on previous successful RRML implementation experiences, where similar TAT were consistently achieved under field conditions [13], providing a practical reference for deployment readiness. These findings align with studies emphasising that structured, hands-on training and mentorship are critical for building proficiency in high-containment laboratory operations [5, 9].

Importantly, the study also identified substantial attrition within the trained expert pool. This reflects the broader challenge faced by many national health systems in retaining highly specialised professionals, a factor that extends beyond the scope of the MOBILISE project. Nevertheless, it underscores the importance of incorporating workforce retention considerations into surge capacity planning and raises important questions regarding the long-term sustainability and resilience of expert pooling approaches for emergency response [10, 11].

A key component of the programme is the ToT approach, designed to create a core group of highly qualified professionals capable of cascading their knowledge and skills to new generations of experts. This cascading model ensures both the widespread adoption of standardised laboratory practices and biosafety standards and the sustainability of the infectious disease response system by maintaining an interconnected network of trained personnel across Europe and Africa. Similar strategies have been reported in other laboratory training programmes, where structured mentorship and repeated field exercises were adopted to consolidate competencies and foster operational resilience [13, 14].

The assessment of both online and in-person training programmes provides important insights into the effectiveness and limitations of the MOBILISE curriculum. The online modules reinforced participants’ baseline knowledge, with measurable improvements in post-test scores, suggesting that even experienced professionals benefit from structured, theory-based instruction. This aligns with previous studies indicating that well-designed e-learning programmes can effectively consolidate knowledge and prepare trainees for practical, high-containment laboratory work [13–15]. The absence of a standardised observational instrument during field validation limits quantification of preparedness gains; future iterations should incorporate structured tools (e.g., validated checklists) alongside expert mentorship.

The in-person training demonstrated that hands-on experience is critical for translating theoretical knowledge into practical competence, particularly in high-risk field settings. Trainees were able to integrate multiple operational aspects such as biosafety, sample handling, molecular diagnostics, and LIMS use, under realistic conditions, highlighting the value of immersive, experiential learning [16]. This approach is consistent with competency-based education (CBE) frameworks, which emphasise the development and assessment of demonstrable skills through performance in real or simulated operational environments rather than solely theoretical instruction [12].

In parallel, the MOBILISE training programme contributes to health workforce surge capacity by strengthening the availability of trained personnel capable of rapid deployment and effective scaling of diagnostic operations during outbreak responses. This aligns with broader concepts of health system resilience and global health security preparedness. The WHO Minimum Operational Standards and Typology for Rapid Response Mobile Laboratories emphasises the need for a skilled, trained and rapidly deployable workforce to ensure operational readiness across deployment cycles and highlights workforce development as a key element for strengthening RRML teams, enabling continuous capacity building in both emergency and non-emergency contexts, while contributing to the reinforcement of national laboratory capacities through structured and standardised training approaches [11, 17, 18].

Specifically, by applying the ToT model, the MOBILISE programme expanded its capacity from an initial group of eight ToTs to a total of 21 experts fully trained to operate the mobile laboratory across Europe and Africa. Despite these achievements, maintaining continuity within the expert pool remains challenging. Field missions revealed that previously trained ToTs were not always available, requiring the recruitment and training of new teams, which underscores the difficulty of retaining highly specialised personnel. In the short term, increasing the number of trained trainers or sharing ToTs in a common pool could be a solution to overcome this bottleneck, but continuity in lab staff remains a major challenge.

Staff shortages continue to pose a major obstacle to sustaining laboratory operations. Surveys of clinical laboratory personnel report high attrition, with over 60% leaving within the first five years, driven by factors such as pursuit of new laboratory positions, family obligations, and retirement [19]. Limited career awareness, non-competitive compensation, and growing demand for laboratory services further reduce the availability of qualified personnel. Addressing these gaps requires promoting laboratory careers, implementing structured training and mentorship, providing clear career development pathways, and fostering early engagement through educational and outreach initiatives [15]. However, formal retention strategies specific to the MOBILISE expert pool have not been developed, as workforce retention falls primarily within the mandate of national institutions rather than the project itself. Nonetheless, the data generated by this study reinforce the importance of national and institutional policies aimed at retaining highly specialised professionals and may inform decision-makers to develop stronger incentives and career opportunities to support the long-term retention of trained personnel.

Looking forward, the expansion of the One Health expert pool is essential to address the increasing frequency and complexity of infectious disease outbreaks driven by climate change and global travel. Incorporating continuous refresher training, scenario-based exercises, and cross-border collaborations can enhance both technical proficiency and adaptability, ensuring that mobile laboratories are staffed with resilient and highly competent teams [20]. An additional key element is the implementation of recurring training opportunities using mobile laboratory platforms, which would allow continuous hands-on skill reinforcement under realistic conditions; however, such activities are often constrained by limited funding outside outbreak periods. Additionally, integrating new diagnostic technologies and expanding the scope of pathogens included in training exercises can further strengthen preparedness, as has been suggested in recent assessments of European outbreak response capacity [6, 21].

Importantly, sustainability of the One Health expert pool beyond the MOBILISE project funding period is supported by its integration within existing national structures. The experts involved are part of the workforce of National Public Health Institutions or National Agencies in their respective countries, where they remain employed and engaged in routine preparedness and emergency response activities. Consequently, their availability is maintained through national surge capacity systems rather than project-dependent funding. Ownership and maintenance of the expert roster are decentralised, with each participating institution responsible for updating information on its own personnel and expertise.

Beyond workforce retention, the sustainability of the expert pool also depends on equitable partnerships and local ownership. Partner institutions actively contributed as equal members of the consortium, participating in the design and implementation of field missions and providing local coordination during deployment activities. The training model was oriented towards capacity strengthening and knowledge transfer rather than dependency, enabling partner countries to independently operate and adapt mobile laboratory workflows within their national systems. The ToT approach further supported this objective by decentralising expertise and promoting local multiplication of skills, with trained Tanzanian partners subsequently contributing to Ebola Bundibugyo outbreak response activities. The expert pool also reflected a balanced gender distribution, with 12 of the 21 trained experts being women.

Although BNITM serves as the custodian of the mobile laboratory, all project partners are entitled to request its deployment during outbreak situations. In the event of international health emergencies, cross-border deployment is facilitated through established collaboration mechanisms and, where necessary, through existing or case-specific bilateral and multilateral agreements between institutions and countries. In Tanzania, the mobile laboratory remained available for an extended period following the field missions and was used independently by the local team for operational activities before its planned return to Europe in accordance with the temporary import permit. Nonetheless, there is ongoing collaboration between BNITM and NPHL in Tanzania. In addition, box-based mobile laboratories already owned by the Tanzanian NPHL complement existing capacity, and efforts are currently underway to acquire a dedicated mobile laboratory similar to the MOBILISE platform under Tanzanian ownership.

## Conclusion

Overall, the MOBILISE training programme demonstrates that targeted, structured, and cascading training approaches are effective in building a skilled, interconnected, and sustainable workforce capable of responding rapidly to high-risk infectious disease scenarios within a One Health framework. Continued investment in such programmes will be crucial for maintaining operational readiness and ensuring a resilient cross-continental infectious disease response system. At the same time, the attrition observed among trained experts highlights the need for future initiatives to better understand and address the factors influencing expert retention within surge workforce models. Developing strategies to preserve institutional knowledge and maintain the availability of highly specialised personnel will be essential for ensuring the long-term sustainability and effectiveness of expert pooling approaches for emergency response.

## Data Availability

All available data is displayed in the manuscript.
